# Association of HPV and EBV in Oral Verrucous Squamous Cell Carcinoma and Oral Verrucous Hyperplasia

**DOI:** 10.1055/s-0041-1735907

**Published:** 2021-12-02

**Authors:** Eakapong Tamboon, Phetmany Sihavong, Nakarin Kitkumthorn, Dusit Bumalee, Tawepong Arayapisit, Puangwan Lapthanasupkul

**Affiliations:** 1 Department of Oral Biology, Faculty of Dentistry, Mahidol University, Bangkok, Thailand; 2 Dental Basic Science Department, Faculty of Dentistry, University of Health Science, Lao P.D.R, Thailand; 3 Department of Oral and Maxillofacial Pathology, Faculty of Dentistry, Mahidol University, Bangkok, Thailand; 4 Department of Anatomy, Faculty of Dentistry, Mahidol University, Bangkok, Thailand

**Keywords:** oral verrucous squamous cell carcinoma, oral verrucous carcinoma, oral verrucous hyperplasia, HPV, EBV

## Abstract

**Objective** Oral verrucous squamous cell carcinoma or oral verrucous carcinoma (OVC) is a rare verrucous variant of oral squamous cell carcinoma (OSCC), which accounts for 2 to 12% of all oral carcinomas. Oral verrucous hyperplasia (OVH) is clinically similar to OVC and has been proposed to be a precursor lesion of OVC. Etiopathogenesis of both lesions is still inconspicuous. Oncogenic viruses such as human papillomavirus (HPV) and Epstein–Barr virus (EBV) have been reported to be associated with some cases of OSCC, and we hypothesized that it may act as a causative agent of these verrucous lesions. This study aimed to investigate frequency of HPV and EBV infections in OVC and OVH.

**Material and Methods** Using polymerase chain reaction (PCR), a total of 35 formalin-fixed paraffin-embedded (FFPE) tissue samples, including 27 OVC samples and 8 OVH samples, were investigated for HPV and EBV infection. HeLa and B95-8 cell lines were used as positive controls of HPV and EBV PCR, respectively.

**Results** All OVC and OVH samples show a positivity to *GAPDH* , whereas neither HPV nor EBV PCR products was detected in both OVC and OVH samples.

**Conclusions** In summary, our study demonstrated that HPV and EBV are not involved in pathogenesis of OVC and OVH. Other etiologic factors contributing to OVC and OVH need to be further clarified.

## Introduction

Oral verrucous squamous cell carcinoma or oral verrucous carcinoma (OVC), a low-grade variant of oral squamous cell carcinoma (OSCC), accounted for approximately 2 to 12% of all oral carcinomas, with approximately 80% 5-year survival rate. 1 2 Etiological factors of OVC are not clarified but may be related with smoking, areca nut chewing, alcohol, and oral microorganisms. 1 3 Furthermore, many studies have demonstrated human papillomavirus (HPV) infection in OVC. Noble-Topham et al showed that HPV infection was detected (48%) in OVC by polymerase chain reaction (PCR), 4 while Fujita et al found HPV infection in 48% of OVC using PCR and 26% of OVC using *in situ* hybridization (ISH). 5 On the other hand, a few studies did not find HPV infection in OVC. 6 7 These findings indicated that the role of HPV in OVC is inconclusive. Epstein–Barr virus (EBV) is another oncogenic virus that is known to be a cause of nasopharyngeal cancer and B-cell lymphoma. 8 A previous study showed that epithelial cells transfected with the LMP1 (latent membrane protein 1) gene in the skin of transgenic mice were able to induce epithelial hyperplasias or neoplasias. This suggested that EBV may cause proliferation of oral squamous epithelium. 9 Additionally, previous reports revealed that EBV might be related with OVC. 10 11 To date, there is limited information regarding EBV infection in OVC.

OVC is defined by a slow-growing tumor, with a tendency of local invasion and rare recurrence. The clinical features of OVC appear as well-demarcated, painless, thick plaque with papillary or verruciform surface projections. This histopathologic feature may be mistaken for benign epithelial hyperplasia. Interestingly, the clinical and histopathological morphologies of OVC are closely similar to oral verrucous hyperplasia (OVH). Both OVC and OVH show similar clinical appearance, and the common oral sites of these lesions are also similar. 12 13

In the present study, OVC and OVH specimens were examined for the presence of HPV and EBV infections by PCR. The result will support whether OVC and OVH are associated with HPV and EBV.

## Material and Methods

### Ethic Approval and Sample Collection

**Fig. 1:**
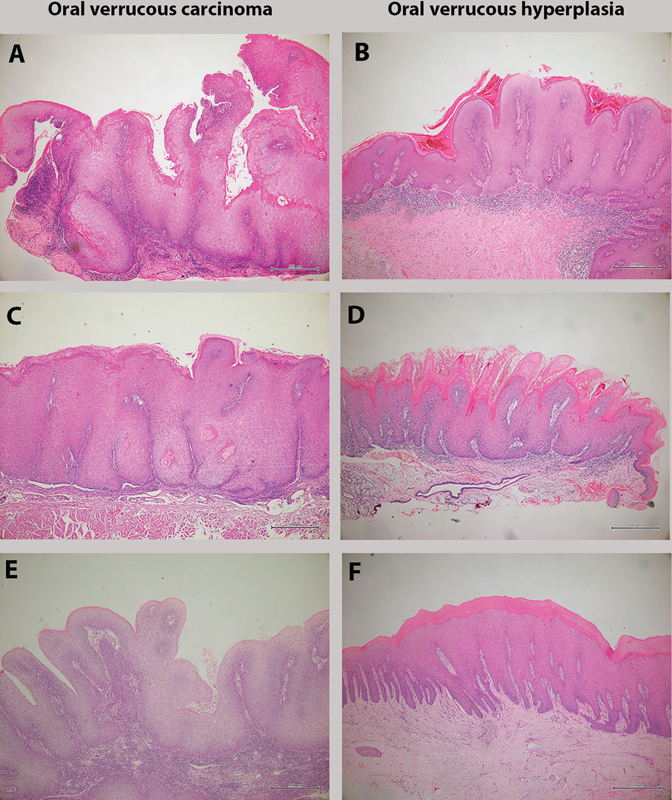
Histopathologic features of oral verrucous squamous cell carcinoma (OVSCC) and oral verrucous hyperplasia (OVH). ( **A, C, E** ) oral verrucous carcinoma (OVC) showing papillary epithelial hyperplasia with bulbous rete ridges pushing into the connective tissue. ( **B, D, F** ) OVH showing epithelial proliferation with verrucous surface elevating from the adjacent normal epithelium.

Ethical consideration of this study was approved by the Institutional Review Board of Faculty of the Dentistry/Faculty of Pharmacy, Mahidol University (COE.No.MU-DT/PY-IRB 2018/060.1312 and 2021/014.0402). Archival pathological specimens of the Department of Oral and Maxillofacial Pathology, Faculty of Dentistry, Mahidol University were assessed, and a total of 35 samples, including 27 cases of OVC and 8 cases of OVH, were retrieved. The clinicopathological information of each case, including age, sex, and tumor location, was collected from pathology requested forms. Histopathological slides of these cases were confirmed by two oral pathologists (P.S. and P.L.). Histopathological diagnosis of OVH and OVC was made, based on criteria recommended by Lin et al. 7 The criteria for a diagnosis of OVH include: 1) epithelial hyperplasia with hyperkeratosis and verruciform surface; 2) the lesional epithelium does not invade the subjacent connective tissue or is elevated compared to the normal adjacent epithelium. The criteria for histopathological diagnosis of OVC were as follows: 1) epithelial hyperplasia with hyperkeratosis, broad blunt rete ridges, and showing no or mild dysplasia; 2) the lesional epithelium pushes into the underlying connective tissue, compared with the normal adjacent epithelium. Histopathological photos of representative OVC and OVH samples were demonstrated in Fig. 1 .

The wax blocks of paraffin-embedded tissue of desired samples were sectioned to obtain tissue sections, approximately three to four ribbons with 10-µm thickness, using a microtome (LEICA RM 2255). The tissue sections were placed into a sterile 1.5 mL microcentrifuge tube for tissue DNA extraction. Genomic DNA extraction was performed, according to the phenol-chloroform protocol previously proposed by Kasperbauer et al. 14 The concentration of obtained DNA was measured using a NanoDrop 2000 spectrophotometer (ND-1000 Spectrophotometer, NanoDrop Technologies, Welmington, DE, USA). The optical density 260/280 ratio being greater than 1.8 is acceptable for DNA purity and PCR. PCR was performed on the extracted DNA using the following three primers: glyceraldehyde 3-phosphate dehydrogenase ( *GAPDH* ) was served as the internal control to test the quality of DNA. GP5 +/GP6+ primers encoding major capsid protein were examined to detect HPV infection. Finally, LMP1 primers encoding EBV latent membrane protein was used to detect EBV infection. The properties of these primers were shown in Table 1 . HeLa cell line (a cervical carcer cell line containing HPV18 DNA) and B95-8 cell line (EBV-producing marmoset B-cell line) were used as positive controls for HPV PCR and EBV PCR, respectively. Distilled water was used as a negative control. The total 10 µl PCR reaction contained 1x PCR buffer (Thermo Scientific, Massachusetts USA), 25 mM MgCl _2_ (Thermo Scientific, Massachusetts, USA), 200 mM dNTPs (Thermo Scientific, Massachusetts, USA), 0.2 mM primers, 0.5 U *Taq* DNA polymerase (Thermo Scientific, Massachusetts, USA), and 50 ng **/** µl of template DNA. All PCR reactions were done in the thermal cycler with the following conditions: 10-minute initial denaturation at 95 °C, 45 cycles of denaturation at 95 °C for 45 seconds, annealing at 55 °C for 45 seconds, extension at 72 °C for 45 seconds, and a final extension at 72 °C for 10 minutes. Following amplification, the PCR products were separated by gel electrophoresis using 1% (w/v) agarose gel in 1x TBE buffer, and then stained with RedSafe Nucleic Acid Staining Solution (Intron, Korea).

**Table 1:** Properties of oligonucleotide primer sequences and conditions for PCR analyses in this study

Detection	Primer	Amplicon size (bp)	Sequence 5′-3′
Internal control ( *GAPDH* )	GAPDH forward	150	CAGCCGCATCTTCTTTTG
	GAPDH reverse		CAACAATATCCACTTTAC
EBV DNA	LMP1 forward	129	CCAGACAGCCAACAATTG
	LMP1 reverse		GGTAGAAGACCCCCTAC
HPV DNA	GP5+	150	TTTGTTACTGTGGTAGATACTAC
	GP6+		CTTATACTAAATGTCAAATAAAAAG

## Results

A total of 35 histopathological samples consisting of 27 specimens of OVC and 8 specimens of OVH were obtained from the Department of Oral and Maxillofacial Pathology, Faculty of Dentistry, Mahidol University. The clinicopathological features of OVC patients were presented in Table 2 . The OVC cases consisted of 21 (77.8%) females and 6 (22.2%) males, with a 3.5:1 female to male ratio, and the mean age of 75.44 years (ranged from 51–103 years). The most common site of the OVC was the buccal mucosa (29.6%), followed by the tongue (18.52%). OVH cases were composed of 6 (75%) females and 2 (25%) males, with a 3:1 female to male ratio, and the mean age of 70.5 years (ranged from 59–84 years). The most common sites of OVH cases were the gingiva (50%) and the buccal mucosa (50%).

**Table 2:** Clinical information of OVC and OVH cases

Characteristics	OVC ( *n* = 27)	OVH ( *n* = 8)
Age, mean (range) years	75.44 (51–103)	70.5 (59–84)
Sex		
Female	21 (77.8%)	6 (75%)
Male	6 (22.2%)	2 (25%)
Location		
Gingiva/alveolar mucosa	5 (18.5%)	4 (50%)
Buccal mucosa	8 (29.6%)	4 (50%)
Lip	3 (11.1%)	0
Tongue	5 (18.5%)	0
Palate	3 (11.1%)	0
Multiple sites	3 a (11.1%)	0

Abbreviations: OVC, oral verrucous carcinoma; OVH, oral verrucous hyperplasia.

a Two cases were located at the lip and buccal mucosa and the other case was found at the gingiva and buccal mucosa.

**Fig. 2:**
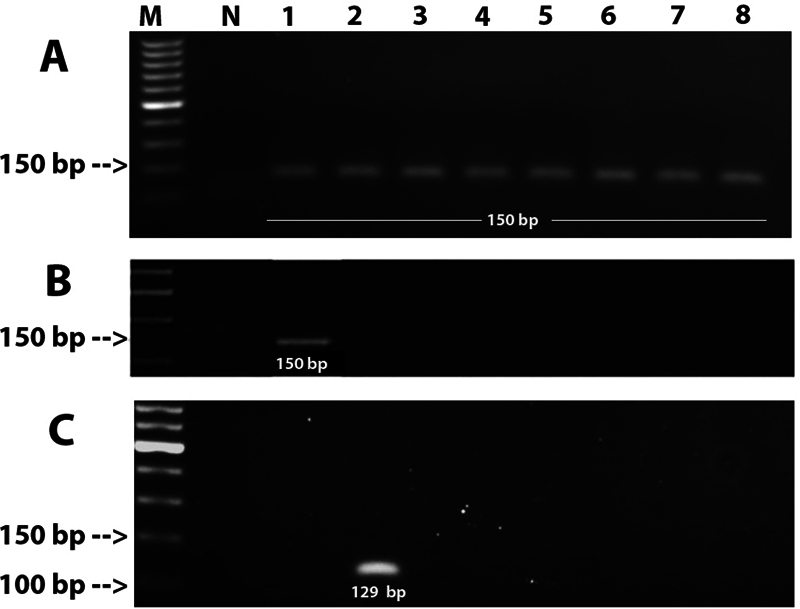
The 1% (w/v) agarose gel electrophoresis of polymerase chain reaction (PCR) products. Lane M: 100 bp ladder; Lane N: negative control; Lane 1 = positive control for human papillomavirus (HPV) (HeLa cell); Lane 2 =positive control for Epstein–Barr virus (EBV) (B95-8 cell); Lane 3-5 = oral verrucous carcinoma (OVC) samples; Lane 6-8 = oral verrucous hyperplasia (OVH) samples. ( **A** ): Representative gel pictures showing polymerase chain reaction (PCR) products positive for *GAPDH* (150 bp) in all samples of OVC and OVH. ( **B** ): Representative gel pictures showing PCR products negative for HPV, only positive control showed amplification (150 bp). ( **C** ): Representative gel pictures showing PCR products negative for EBV, only positive control showed amplification (129bp).

The DNA fragments of human *GAPDH* were successfully amplified in all samples of OVC and OVH in our study. In addition, internal control amplifications were also negative. Our study found that all 35 samples (100%) of OVC and OVH were negative for both HPV and EBV infections. Representatives of the PCR amplification for each gene fragments were shown in Fig. 2 .

## Discussion

Our results demonstrating an absence of HPV and EBV DNA in OVC and OVH is surprising and contradicts our hypothesis that these viral infections would take a role in the promotion of epithelial verrucous proliferation is some OVC or OVH cases. Regarding the previous studies ( Table 3 ), the role of HPV in these oral verrucous lesions has been inconclusive. For OVH, only a very few studies investigating HPV in OVH and questionable results were found. For instance, Shroyer and Greer showed HPV DNA detected in 28.5% of OVH, 15 whereas no HPV DNA was found in OVH from the studies conducted by del Pino et al. 16 In addition, Lin et al found very low expression of HPV 16/18 E6 protein (0.3% mean labeling index) in 30 OVH samples. 7 Our results further support that HPV may have no role in the development of OVH.

**Table 3:** Overview of previous studies investigating HPV in OVC and OVH

Reference	Number and type of samples	Method of HPV detection (primer)	Detected HPV types (overall HPV prevalence in %)
Shroyer and Greer 15	- 14 OVH - 3 OVC (FFPE)	- ISH (31/33/35) - PCR (specific primer for HPV-type 16)	- 16 (28.5%) by PCR in OVH - 16, 31/33/35 (28.5%) by ISH in OVH - No HPV infection in OVC was found.
Noble-Topham et al 4	- 25 OVC (FFPE)	- PCR (6b/11,16,18 primers)	- 6/11, 11, 16, 18, 16/18 (48%)
Shroyer and Greer 18	- 17 OVC (FFPE)	- PCR and DNA slot-blot hybridization - ISH	- 7/17 (41%) - 6/11, 6/18, 31/33/35
Mitsuishi et al 17	-5 OVC	- PCR, sequence analysis and restriction fragment length polymorphism	- 20 (74.8%)
Fujita et al 5	- 23 OVC (FFPE)	- PCR (SPF primer) - ISH (18, 6, 74, 11, 33)	- 18, 6, 74, 11, 33 (48%) by PCR - 6, 8/18, 6/18/74 (26%) By ISH
Saghravanian et al 19	- 21 OVC (FFPE)	- PCR (GP5/GP6)	- 16/18 (14.3%)
de Spindula-Filho 6	- 8 OVC (FFPE)	- PCR (GP5/GP6)	- No HPV infection in OVC
Lin et al 7	- 48 OVC - 30 OVH (FFPE)	- IHC (16/18 E6 protein)	- Very low labeling indices of HPV 16/18 E6 protein in both OVC (0.5%) and OVH (0.3%)
del Pino et al 16	- 5 OVC - 1 OVH (FFPE)	- PCR (GP5/GP6)	- Only 1 case (20%) in OVC - No HPV infection in OVH
Stokes et al 20	- 7 OVC (FFPE)	- PCR (GP5/GP6) - ISH (1R6)	- Only 1 case (14.28%) by PCR - HPV-type 16 (14.28%) by ISH
Sritippho et al 21	- 4 OVC (FFPE)	- Real-time PCR (specific primer for HPV-type 16 and 18)	- Only 1 case (25%) - Coinfection of HPV-16/18

Abbreviations: FFPE, formalin-fixed paraffin-embedded; HPV, human papillomavirus virus; ISH, *in situ* hybridization; OVC, oral verrucous carcinoma; OVH, oral verrucous hyperplasia; PCR, polymerase chain reaction.

HPV detection in OVC is also highly controversial. One previous study demonstrated that HPV DNA was detected in all lip verrucous carcinoma (100%), using PCR with sequence analysis and restriction fragment length polymorphism analysis. However, only five samples were investigated in their study. 17 Several studies from USA, UK, Iran, Canada, and Japan have shown considerably high prevalence of HPV infection (14.28–48%) in OVC. These studies conducted a considerably large cohort of OVC, ranging from 7 to 25 OVC samples. 4 5 18 19 20 On the other hand, other studies revealed no HPV infection in OVC. 6 7 15 In Thailand, although a prior study reported that one (25%) of a total four OVC cases showed coinfection of HPV-16/18, 21 our finding with a larger cohort of OVC (27 cases) suggests that HPV has no relation with OVCs in Thai patients. Taken together, these discrepant findings may be due to differences in ethnicity, and further study is required to elucidate. It is worth noting that the negative relationship between HPV and verrucous carcinoma has also been reported in verrucous carcinoma at the other sites, including vulva and penis. 22 23 24 Similar to OVC, both vulvar and penile verrucous carcinoma are predominantly found in elderly, and show the same clinical and histological features. 24 25 These observations point out that a relationship between verrucous carcinoma and HPV may not be strong.

With regard to EBV infection, only a few studies investigated EBV in OVC, and very few cases were performed in those studies. 11 26 To our knowledge, study of EBV infection in OVH is not found in the English literature. We observed no participation of EBV in 27 OVCs and 8 OVHs. The absence of EBV relation in OVC corresponded to a previous study, demonstrating no EBV DNA in 4 OVC cases using conventional PCR. 26 Nevertheless, the other two studies found EBV infection in one case of OVC using PCR. 10 11 EBV is a known cause of nasopharyngeal carcinoma and is also associated with several oral neoplasms including B-cell lymphomas and lymphoepithelial carcinoma. 8 Several studies previously investigated the possible implication of EBV in OSCC, the common oral malignancy often associated with OVC, but controversial results have been found. 27 28 29

The pathogenesis of these verrucous lesions may be contributed to other factors. The etiology of OVC is complex and strong associations between OVC and alcohol consumption, smoking, areca nut chewing, and oral microbiota have been found. In addition, OVC also shows an association with poor prosthesis, earlier injuries and chronic inflammation, or may result from worsening of premalignant lesions such as oral verrucous leukoplakia. 3 Previously, a subset of OVH has been shown to be associated with a long duration of tobacco and lime quid placement in the buccal vestibule. 30

Numerous studies investigated HPV and EBV infections using PCR method, a laboratory technique used to synthesize the multiple copies of a fragment of DNA. This technique is widely accepted due to its more sensitivity and reliability. It has been proposed that in comparison to DNA isolated from fresh or frozen tissue, the quality of DNA extracted from formalin-fixed paraffin-embedded (FFPE) was inferior and may lead to the negative PCR result. 31 Nonetheless, our study found no HPV and EBV DNA in both OVC and OVH; DNA amplification for *GAPDH* , a housekeeping gene, was successful in all cases. This indicated that DNA of our samples were good quality for DNA amplification.

## Conclusion

In conclusion, our results do not support HPV and EBV as a potential cause for the development of OVH and OVC in Thai patients. Therefore, further studies may be needed to elucidate the etiology of these lesions.
